# Dimerization and DNA-dependent aggregation of the *Escherichia coli* nucleoid protein and chaperone CbpA

**DOI:** 10.1111/j.1365-2958.2010.07292.x

**Published:** 2010-07-30

**Authors:** Sarah Cosgriff, Kiran Chintakayala, Ya Tsz A Chim, Xinyong Chen, Stephanie Allen, Andrew L Lovering, David C Grainger

**Affiliations:** 1 Department of Biological Sciences, University of WarwickCoventry CV4 7AL, UK; 2 Laboratory of Biophysics and Surface Analysis, School of Pharmacy, University of Nottingham, University ParkNottingham NG7 2RD, UK; 3 School of Biosciences, University of BirminghamEdgbaston, Birmingham, B15 2TT, UK

## Abstract

The *Escherichia coli* curved DNA-binding protein A (CbpA) is a nucleoid-associated DNA-binding factor and chaperone that is expressed at high levels as cells enter stationary phase. Using a combination of genetics, biochemistry, structural modelling and single-molecule atomic force microscopy we have examined dimerization of, and DNA binding by, CbpA. Our data show that CbpA dimerization is driven by a hydrophobic surface comprising amino acid side chains W287 and L290 located on the same side of an α helix close to the C-terminus of CbpA. Derivatives of CbpA that are unable to dimerize are also unable to bind DNA. Free in solution, CbpA can exist as either a monomer or dimer. However, when bound to DNA, CbpA forms large aggregates that can protect DNA from degradation by nucleases. These CbpA–DNA aggregates are similar in morphology to protein–DNA complexes formed by the DNA-binding protein from starved cells (Dps), the only other stationary phase-specific nucleoid protein. Conversely, protein–DNA complexes formed by Fis, the major growth phase nucleoid protein, have a markedly different appearance.

## Introduction

Bacterial chromosomes are organized by a combination of supercoiling, transcription and nucleoid-associated DNA-binding proteins (Jin and Cabrera, 2006; Zimmerman, 2006; Dorman, 2009). By modulating these activities the bacterial cell can change the way in which the chromosome is structured in response to environmental signals (Kim *et al*., 2004; Ohniwa *et al*., 2006; Dillon and Dorman 2010). In *Escherichia coli*, a particularly striking change in nucleoid structure occurs as cells enter stationary phase; the nucleoid adopts a super compact conformation and it is believed that this is a mechanism via which the cell can protect its DNA in harsh conditions (Kim *et al*., 2004). Nucleoid-associated proteins are believed to play a key role in driving super compaction of the nucleoid and, of all the nucleoid-associated proteins, only two are specifically induced as cells approach stationary phase: the DNA-binding protein from starved cells (Dps) (Almirón *et al*., 1992) and curved DNA-binding protein A (CbpA) (Yamada *et al*., 1990; Ueguchi *et al*., 1994).

The DNA-binding protein from starved cells is the most abundant nucleoid protein in stationary phase *E. coli*, present at up to 100 000 copies per cell, and is evenly distributed across the nucleoid (Azam *et al*., 1999; 2000;). First identified as a protein with a stationary phase-specific expression profile, Dps binds DNA with no apparent sequence specificity and protects cells from treatment with H_2_O_2_ (Almirón *et al*., 1992). Structural analysis of Dps revealed similar protein folds to those found in bacterioferritin (Grant *et al*., 1998). Thus, it has been proposed that Dps can sequester Fe^2+^, preventing the generation of free radicals via Fenton chemistry (Grant *et al*., 1998). The Dps protein is also thought to protect DNA from damage by physically shielding it from harmful agents. Thus, electron microscopy was used to visualize Dps and Dps–DNA complexes and showed that Dps forms uniformly organized aggregates when bound to DNA (Wolf *et al*., 1999). Dps–DNA aggregates were observed in single-molecule atomic force microscopy (AFM) experiments although the organization of Dps molecules within the aggregates could not be determined (Ceci *et al*., 2004). Consistent with these observations, it has been shown that compaction of the bacterial chromosome in stationary phase requires Dps (Wolf *et al*., 1999).

**Fig. 1:**
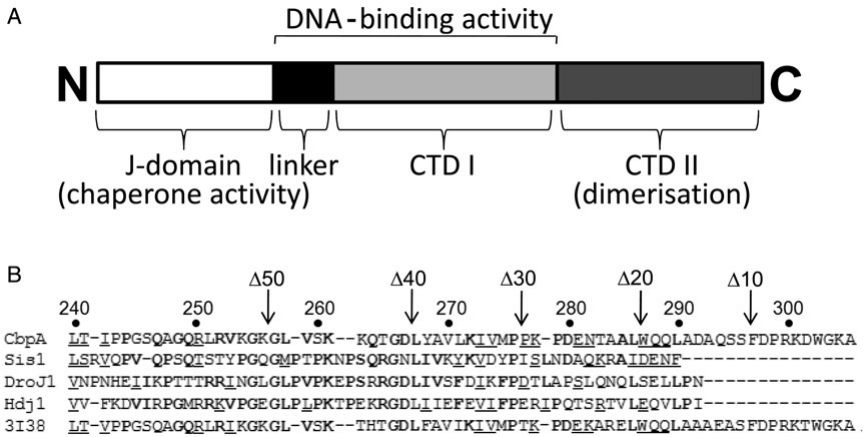
The *Escherichia coli* CbpA protein. Panel A shows a schematic representation of the CbpA protein. Panel B shows an alignment of the C-terminal ends of CbpA, Sis1, DroJ1, Hdj1 and 3I38 (based on the alignments of Bird *et al*., 2006). Positions at which we have introduced C-terminal truncations in the CbpA protein are shown by arrows and are labelled with the name of the protein construct.

Curved DNA-binding protein A was originally discovered 20 years ago as a protein present in *E. coli* cell extracts that preferentially bound to curved DNA (Yamada *et al*., 1990; Ueguchi *et al*., 1994). It has subsequently been shown that CbpA accumulates to between 3000 and 15 000 molecules per cell in stationary phase and forms foci within the nucleoid (Azam *et al*., 1999; 2000; Chenoweth and Wickner, 2008). The N-terminal 75 amino acids of CbpA form a ‘J-domain’, found in chaperone proteins, joined to two C-terminal domains (CTD I & CTD II) by a flexible linker (Fig. 1A). The DNA-binding activity of CbpA locates to the linker and CTD I domain while the 104 amino acid CTD II domain is required for dimerization (Fig. 1; Bird *et al*., 2006). In a *dnaJ* background, disruption of the *cbpA* gene reduces cell viability 10-fold and produces cells with an altered morphology and multiple nucleoids (Ueguchi *et al*., 1995; Chenoweth *et al*., 2007). The chaperone activity of CbpA is repressed by DNA binding and by association of CbpA with CbpM, a protein that is coexpressed with CbpA (Chae *et al*., 2004). The affinity of CbpA for DNA is similar to that observed for other nucleoid proteins and, like Dps, CbpA expression is dependent on σ38-associated RNA polymerase (Chenoweth and Wickner, 2008; Grainger *et al*., 2008).

Here we have used a combination of genetics, biochemistry and structural modelling to study dimerization and oligomerization of CbpA in the presence and absence of DNA. Previous work has shown that CbpA derivatives lacking the CTD II domain are unable to dimerize (Bird *et al*., 2006) and similar conclusions have been reached for the CbpA yeast homologue Sis1 (Sha *et al*., 2000). We show that key CbpA dimerization determinants are located on an α helix located 10 amino acids from the C-terminus of the protein. Residues W287 and L290, located on the same side of this helix, participate directly in dimerization by creating a hydrophobic surface. CbpA derivatives that are unable to dimerize have a greatly reduced affinity for DNA but can still interact with CbpM. Using AFM we show that, while CbpA forms either a monomer or dimer in solution, it forms aggregates with DNA, irrespective of the topological state of the DNA. These CbpA–DNA aggregates have a remarkably similar appearance to Dps–DNA complexes observed previously but differ substantially to DNA complexes formed by the major growth phase nucleoid protein Fis.

## Results and discussion

### A two-hybrid system can be used to detect CbpA–CbpA and CbpA–CbpM interactions

**Fig. 2:**
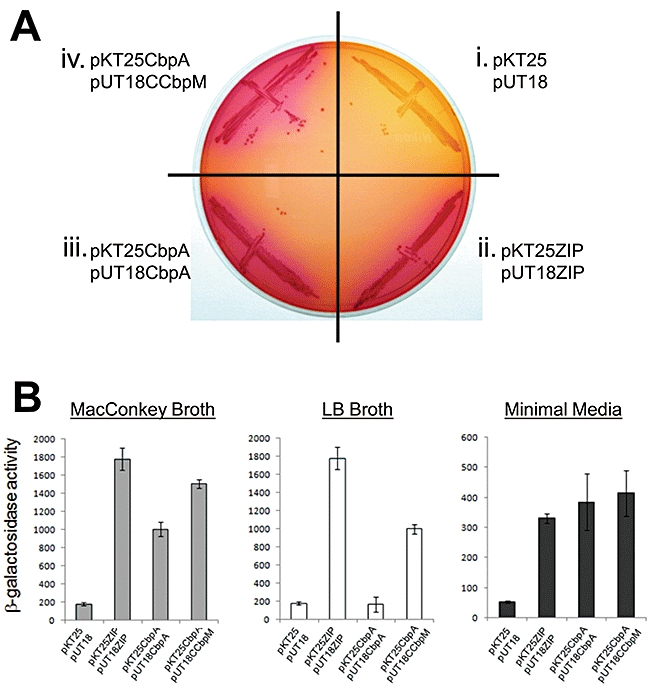
Detection of CbpA–CbpA and CbpA–CbpM interactions *in vivo*. Panel A shows *cyaA*- BTH101 cells growing on a MacConkey Agar plate. The cells carry different derivatives of the pKT25 and pUT18 plasmid that encode T18 and T25, independently folding domains of adenylyl cyclase. Cells expressing T18 and T25 only have a *lac*- phenotype (i). Fusion of a leucine zipper (ii), CbpA (iii), or CbpA and CbpM, respectively (iv), to the T25 and T18 adenylate cyclase domains results in a *lac*+ (red) phenotype. Panel B shows bar charts illustrating β-galactosidase activities in BTH101 cells, carrying the different pKT25 and pUT18 derivatives, in three growth medium.

Biochemical studies with purified proteins have shown that CbpA can dimerize and can form a complex with CbpM (Bird *et al*., 2006). To measure CbpA–CbpA and CbpA–CbpM interactions *in vivo*, we used the Bacterial two-hybrid (BACTH) assay system, which relies on the fact that the *E. coli cyaA*- strain BTH101 is unable to produce cAMP and thus has a *lac*- phenotype (Karimova *et al*., 1998). This strain can be transformed with plasmids pUT18 (or pUT18C) and pKT25 that encode two independently folding domains (T18 and T25) of the *Bordetella pertussis* adenylyl cyclase enzyme. When these plasmids are modified, so that T18 and T25 are fused to proteins that interact with each other, a *lac*+ phenotype is conferred upon the cell. Thus, protein–protein interactions can be quantified by measuring β-galactosidase activity. Figure 2A shows a MacConkey lactose indicator plate on which BTH101 cells, transformed with pKT25 & pUT18 or derivatives, have been grown. Cells containing pKT25 and pUT18 have a *lac*- (white) phenotype while cells containing pKT25-ZIP and pUT18-ZIP (encoding T25 and T18 proteins fused to a leucine zipper) have a *lac*+ (red) phenotype. A *lac*+ phenotype was also observed for BTH101 cells transformed with pKT25CbpA in combination with either pUT18CbpA or pUT18CCbpM. Thus, both CbpA–CbpA and CbpA–CbpM interactions are detected by the BACTH assay.

To quantify the CbpA–CbpA and CbpA–CbpM interactions the four sets of transformants shown in Fig. 2A were grown overnight in liquid culture and then β-galactosidase levels in the cultures were determined. Surprisingly, the results of these measurements varied dramatically depending on the growth medium used (Fig. 2B). Thus, while we were able to detect the CbpA–CbpM interaction in all conditions, the CbpA–CbpA interaction could be detected in MacConkey broth and Minimal Medium but not LB broth. Because the most robust signals for CbpA dimerization and the CbpA–CbpM interaction were observed in MacConkey broth, this medium was used for all further experiments.

### Effect of C-terminal truncations on CbpA dimerization

**Fig. 3:**
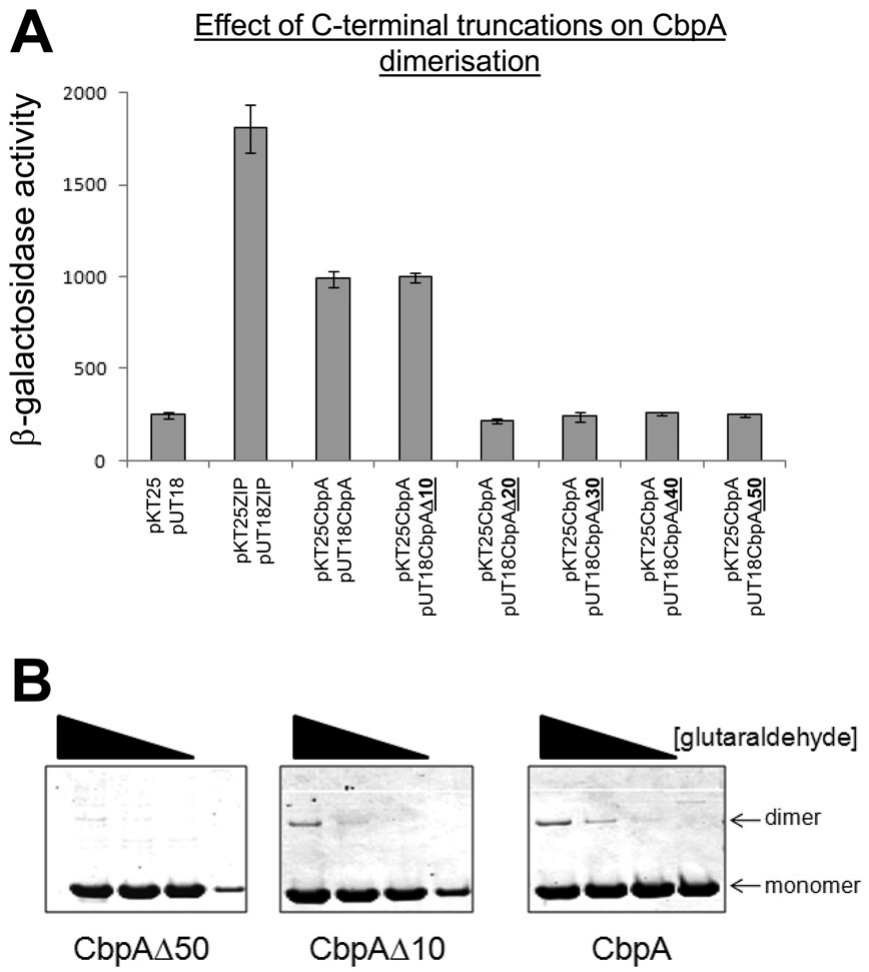
Effect of C-terminal truncations on CbpA dimerization *in vivo* and *in vitro*. Panel A shows β-galactosidase activities for BTH101 cells carrying derivatives of pKT25 and pUT18. Plasmids encoding versions of CbpA with C-terminal deletions (Δ) of 10, 20, 30, 40 or 50 amino acids are highlighted by bold typeface and are underscored. Panel B shows purified CbpA and the C-terminally truncated CbpAΔ10 and CbpAΔ50 derivatives run an SDS-PAGE gel. Proteins (5 ng) were treated with 0.001%, 0.002% or 0.004% glutaraldehyde before loading. A small amount of untreated protein was also run as a ‘marker’ for the CbpA monomer. Monomeric and dimeric forms of CbpA are indicated.

We next used the BACTH system to identify regions of CbpA required for dimerization. Previously, it has been shown that CbpA derivatives lacking the entire 104 amino acid CTD II cannot dimerize. Moreover, structural analysis showed that Sis1 reside D349, equivalent to CbpA residue W287 (Fig. 1B) participated directly in dimerization (Sha *et al*., 2000). Thus, derivatives of pUT18, encoding CbpA lacking 10, 20, 30, 40 or 50 amino acids from the C-terminus were constructed (named CbpAΔ10, Δ20, Δ30, Δ40 and Δ50, respectively; Fig. 1B). Figure 3A shows the results of a BACTH experiment designed to investigate the effects of these truncations on CbpA–CbpA interactions. The data show that while CbpAΔ10 can interact with wild-type CbpA, the signal due to CbpA dimerization is abolished for the Δ20, Δ30 Δ40 and Δ50 CbpA derivatives. To confirm this, dimerization of the purified CbpA, CbpAΔ10 and CbpAΔ50 proteins was also tested *in vitro* by glutaraldehyde cross-linking (Fig. 3B). As expected, CbpA and CbpΔ10 are able to dimerize while dimerization of CbpAΔ50 is reduced.

### Alanine scanning mutagenesis of CbpAΔ10

**Fig. 4:**
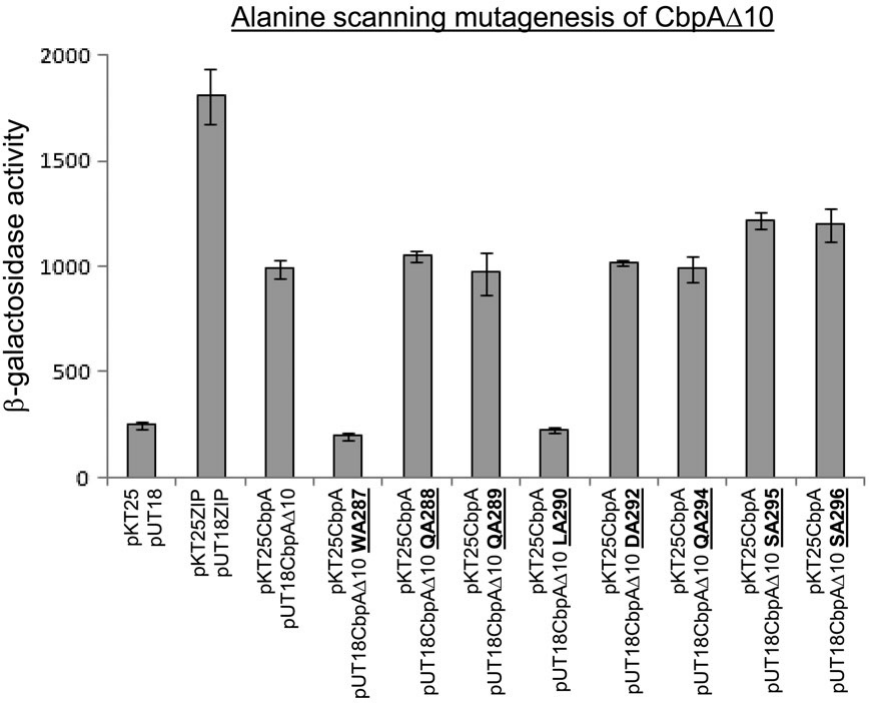
Alanine scanning mutagenesis of CbpAΔ10. The graph shows β-galactosidase activities for BTH101 cells carrying derivatives of pKT25 and pUT18. Plasmids encoding CbpAΔ10 with single alanine substitutions are highlighted with bold typeface and are underscored.

The data in Fig. 3 show that amino acid determinants required for CbpA dimerization must be located somewhere between CbpA residues 287 and 296 (i.e. between 10 and 20 residues from the C-terminus of the protein). Thus, alanine substitutions were made at codons 287, 288, 289, 290, 292, 294, 295 and 296 of *cbpA*Δ10 cloned in the pUT18 vector (we worked with *cbpA*Δ10, rather than full-length *cbpA*, to facilitate mutagenesis). Note that CbpA already has alanine residues at positions 291 and 293 and these positions were therefore not altered. The pUT18CbpAΔ10 derivatives were then used to transform BTH101 cells harbouring pKT25CbpA and β-galactosidase activities were measured in overnight cultures of the transformants. The data are shown in Fig. 4 alongside data from control experiments. The results show that two alanine substitutions, WA287 and LA290, substantially reduce levels of β-galactosidase activity, while all of the other alanine substitutions that we tested had no effect. Thus, WA287 and LA290 derivatives of CbpAΔ10 are unable to interact with wild-type CbpA.

### CbpAΔ10 derivatives unable to dimerize can interact with CbpM

**Fig. 5:**
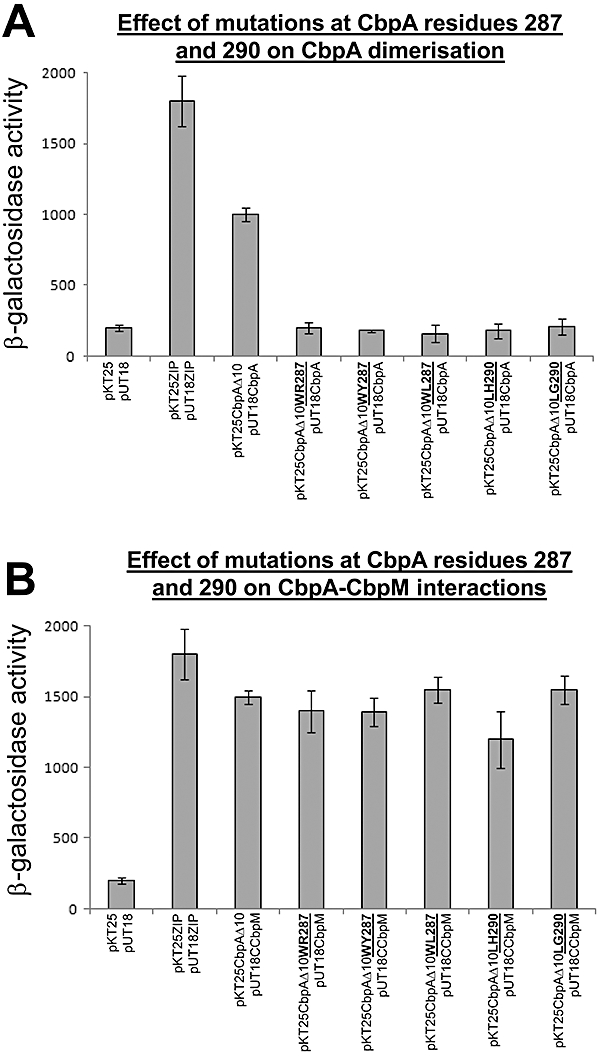
Dimerization defective CbpAΔ10 derivatives can interact with CbpM. The figure shows β-galactosidase activities for BTH101 cells carrying derivatives of pKT25 and pUT18. Plasmids encoding CbpAΔ10 with point mutations at amino acids 287 or 290 are highlighted with bold typeface and are underscored. Panel A shows the ability of these CbpAΔ10 derivatives to dimerize and Panel B shows data for their interaction with CbpM.

We found that the CbpAΔ10 WA287 and LA290 substitutions reduced, but did not abolish, the ability of CbpAΔ10 to interact with CbpM (Fig. S1; note that, in these experiments, *cbpA* alleles were transferred to pKT25). We therefore assume that the WA287 and LA290 substitutions must induce mild perturbations in the CbpAΔ10 structure that reduce the ability of CbpAΔ10 to interact with CbpM. We reasoned that it may be possible to ‘repair’ the CbpAΔ10–CbpM interaction by introducing amino acid side chains other than alanine at positions 287 and 290. Moreover, these CbpAΔ10 derivatives should remain unable to dimerize if W287 and L290 truly form the dimerization surface of the protein. Thus, we randomly mutagenized codons 287 and 290 of *cbpA*Δ10 and selected 114 *cbpA*Δ10 derivatives for further study. Because there is redundancy in the genetic code, we isolated many amino acid substitutions numerous times. Most of these changes resulted in misfolded proteins unable to dimerize or interact with CbpM (data not shown). However, we identified five dimerization defective CbpAΔ10 derivatives (WR287, WY287, WL287, LH290 & LG290) that were able to interact with CbpM (compare Fig. 5A and B). As they can interact with CbpM, these CbpAΔ10 derivatives must be correctly folded but lack key determinants for dimerization. Importantly, we discovered no substitutions that permitted both CbpA dimerization and CbpA–CbpM interactions.

### Interactions of CbpAΔ10 WL287 *in vitro*

**Fig. 6:**
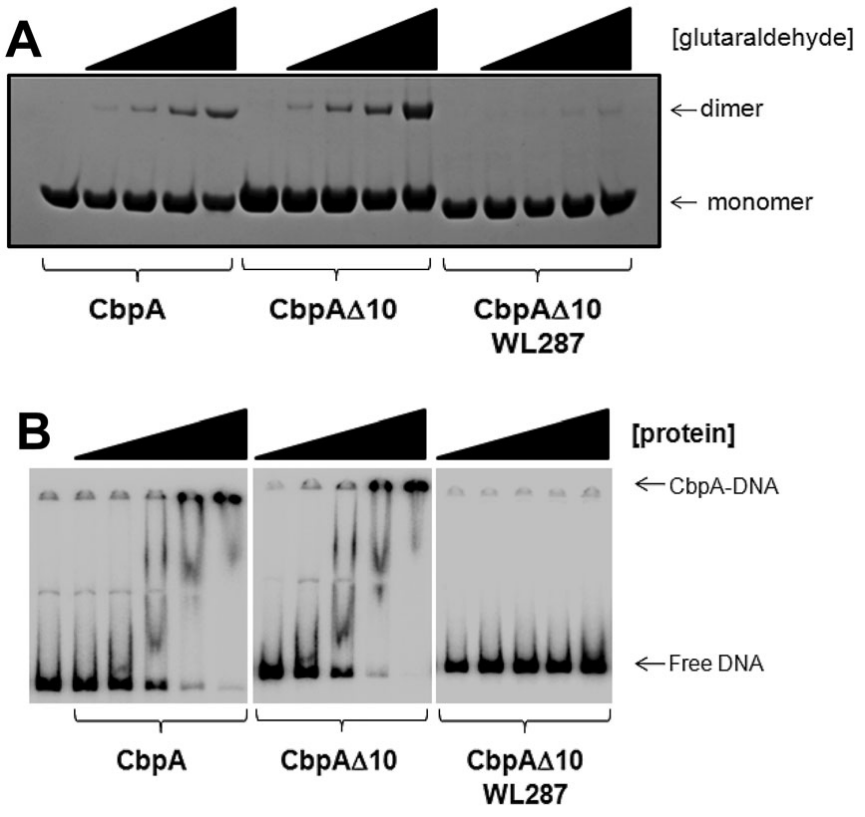
Dimerization and DNA-binding properties of CbpAΔ10 WL287 *in vitro*. Panel A shows purified CbpA, CbpAΔ10 and CbpAΔ10 WL287 run an SDS-PAGE gel. Proteins (5 ng) were treated with 0%, 0.002%, 0.004%, 0.008% or 0.016% glutaraldehyde before loading. Monomeric and dimeric forms of CbpA are indicated. Panel B shows phosphor imager scans of electrophoretic mobility shift assays to detect the binding of CbpA and derivatives to DNA. Proteins were added at a final concentration of 50, 100, 200, 400 or 800 nM and 5 ng of DNA was present in the incubations.

We purified CbpAΔ10 WL287 (Fig. 5) for further study *in vitro*. First, we confirmed the results of our BACTH analysis using glutaraldehyde cross-linking. As expected, while CbpA and the Δ10 derivative were able to dimerize *in vitro*, CbpAΔ10 WL287 was not (Fig. 6A). We then tested the ability of these CbpA derivatives to bind to DNA using electrophoretic mobility shift assays. Figure 6B shows that, while wild-type CbpA and CbpAΔ10 bind DNA with the same affinity, CbpAΔ10 WL287 has a greatly reduced affinity for DNA.

### Single-molecule AFM analysis of CbpA and CbpA–DNA complexes

**Fig. 7:**
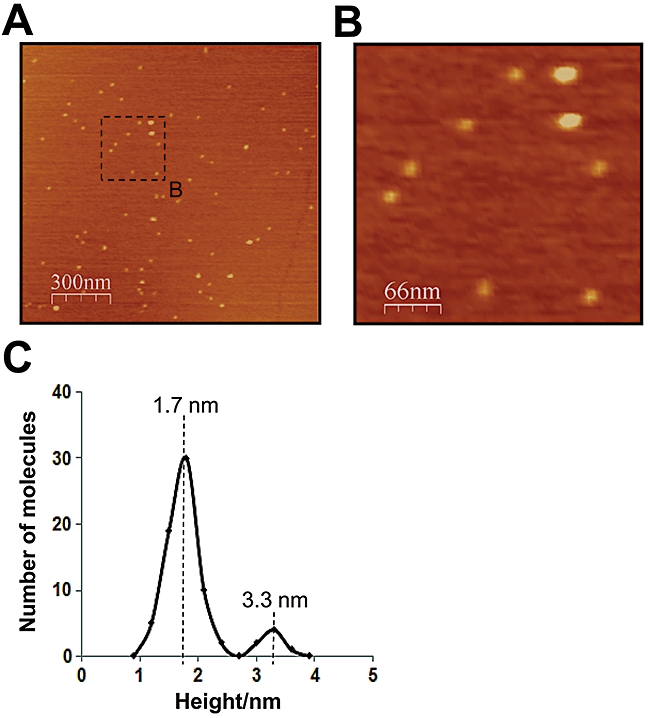
AFM analysis of purified CbpA protein. Panel A shows a AFM generated image of a 1.5 µm × 1.5 µm region of a freshly cleaved 0.5 cm^2^ mica surface on which 20 µl of 100 nM purified CbpA protein had been deposited. The region highlighted by a broken line and labelled ‘B’ is expanded in panel B. Panel C shows a histogram of height measurements for > 100 surface bound particles of CbpA protein.

CbpA–DNA complexes appear as a smear in gel shift assays and, at higher CbpA concentrations, aggregate in the wells of the gel (Fig. 6B). One possibility is that this is due to oligomerization of CbpA across the DNA. This is in sharp contrast to our glutaraldehyde cross-linking (Figs 3B and 6A) and a native PAGE analysis of CbpA interactions (Fig. S2) that show CbpA predominantly exists as a monomer or dimer in the absence of DNA. We were intrigued by the possibility that CbpA may form aggregates with DNA as this is also a property of Dps (Wolf *et al*., 1999; Ceci *et al*., 2004). To investigate this further we used AFM to visualize CbpA and CbpA–DNA complexes at the single-molecule level. Figure 7 shows AFM images of CbpA protein deposited on a sheet of mica. Consistent with our glutaraldehyde cross-linking experiments we observed no large aggregates of CbpA (Fig. 7A). The images show that CbpA particles exist in two forms, one larger than the other (Fig. 7B). Detailed measurements show that the most abundant CbpA particle is ∼1.7 nm in height while the second, less frequent, CbpA particle is ∼3.3 nm in height (Fig. 7C). Thus, these particles likely represent monomers and dimers of CbpA.

**Fig. 8:**
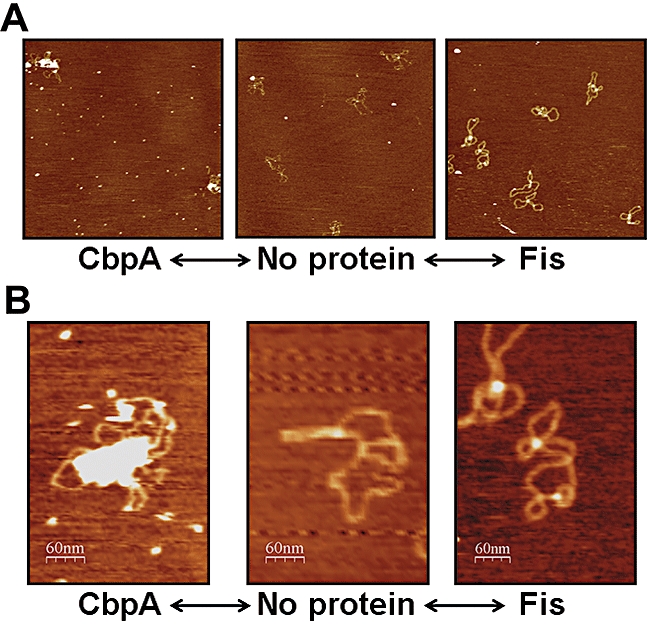
AFM analysis of CbpA–DNA and Fis–DNA complexes. Panel A shows AFM generated images of 1.4 µm × 1.4 µm regions of three separate freshly cleaved 0.5 cm^2^ mica surfaces. Each surface was pre-incubated with 20 µl solutions of plasmid DNA (23 ng) either in the absence of protein or in the presence of CbpA or Fis (both added at a final concentration of 100 nM). Panel B shows close up images of plasmid DNA, CbpA–DNA or Fis–DNA complexes imaged using AFM.

To investigate the effect of DNA on the aggregation state of CbpA, supercoiled pSR plasmid was incubated with CbpA prior to AFM analysis. As a control, we also performed parallel incubations with the same plasmid and an equivalent amount of Fis protein. Note that Fis and CbpA are nucleoid proteins abundant in rapidly growing and starved cells respectively (Azam *et al*., 1999). The results of our analysis are shown in Fig. 8A. In the absence of protein the supercoiled plasmid DNA appears as a contorted circular structure. Fis was found to bind at two or three discrete locations across the plasmid and formed synapse like structures, consistent with Fis-mediated stabilization of DNA cross-points (Schneider *et al*., 2001). The CbpA–DNA complexes had a strikingly different morphology to both the free DNA and the Fis–DNA complexes. The data clearly show that CbpA forms large aggregates (> 60 nm in diameter) when bound with DNA. These aggregates either contain multiple plasmids or adhere poorly to the mica surface because, despite using the same amount of DNA, they are observed less frequently than the free DNA or Fis–DNA complexes. Close up views of single complexes are shown in Fig. 8B. In AFM experiments using a 10-fold higher concentration of CbpA we observed no unbound regions of DNA and we assume that this is because all DNA had been incorporated into protein–DNA aggregates (Fig. S3). Aggregation of CbpA also occurred with liner DNA fragments (Fig. S4).

### CbpA can protect plasmid DNA from degradation by nucleases

**Fig. 9:**
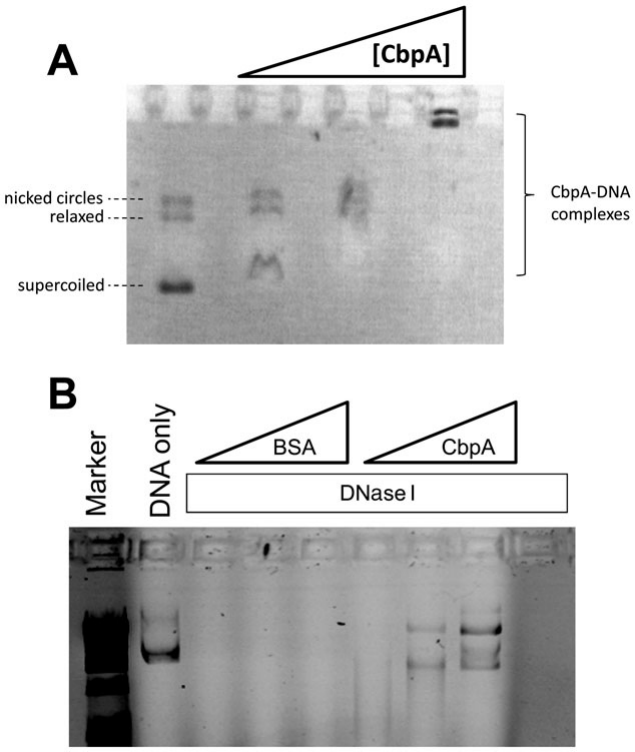
CbpA protects plasmid DNA from degradation by nucleases. A. The panel shows naked plasmid and complexes with CbpA run on a 1% agarose gel. Plasmid (30 ng) was pre-incubated with 0, 0.5, 1.0, or 2.0 µM CbpA. Note that only every other lane has been loaded on the gel. B. The panel shows plasmid run on a 1% agarose gel. Plasmids (77 ng) were treated with different combinations of DNase I, BSA (1, 2 or 3 µM) and CbpA (1, 2 or 3 µM).

Our AFM analysis suggests that CbpA can interact with large tracts of plasmid DNA and incorporates them into aggregates of protein and nucleic acid. We also note that, in Fig. 8, some regions of DNA remain unbound by CbpA. These unbound regions of DNA may occur randomly or may be specific regions of the DNA with a low affinity for CbpA. We reasoned that, if CbpA bound only to specific regions of the plasmid, then unbound regions would be much more sensitive to degradation by nucleases. Alternatively, if CbpA binds non-specifically, it should be possible for CbpA to completely protect plasmids from nuclease degradation. To test these possibilities we first used EMSA analysis to check that CbpA did not specifically target different topological forms of plasmid DNA. The results in Fig. 9A show that CbpA has a similar affinity for all plasmid isoforms. Figure 9B shows the results of an experiment where the same plasmid was treated with DNase I either in the presence or the absence of CbpA. The analysis shows that CbpA can completely protect plasmid DNA from DNase I digestion.

## Conclusions

**Fig. 10:**
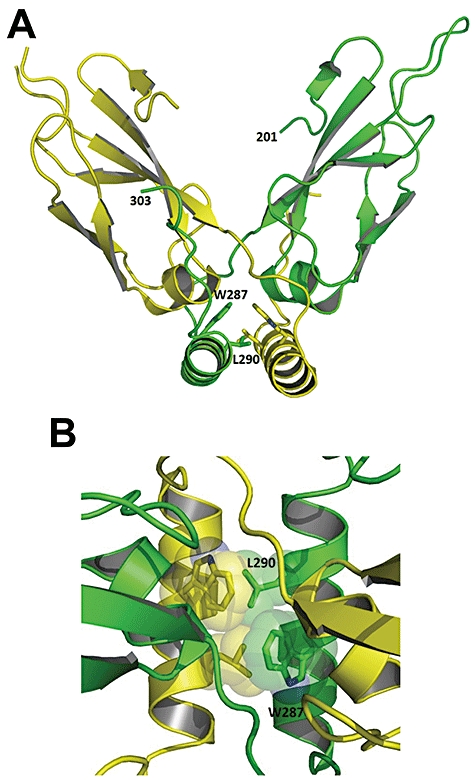
Structural model of the CbpA CTD II dimer. A. The CbpA CTD II monomers are shown in yellow and green respectively. B. Amino acid side chains W287 and L290 are highlighted and form a hydrophobic surface that is buried at the dimerization interface.

The *E. coli* nucleoid undergoes major conformational changes as cells progress through a period of rapid growth, exhaust their supply of nutrients and enter stationary phase (Kim *et al*., 2004). These changes are driven by fluctuations in the available pool of nucleoid-associated proteins (Ohniwa *et al*., 2006). Thus, the major growth phase nucleoid protein Fis is replaced by Dps and CbpA in stationary phase. This is significant because Fis binds to specific DNA target sites while Dps and CbpA bind DNA with little or no sequence specificity. Here we have investigated the dimerization, DNA binding and aggregation properties of CbpA. While this manuscript was in preparation, a structure for residues 200–302 of *Klebsiella pneumonia* CbpA was deposited in the Protein Data Bank (code 3I38, Midwest Center for Structural Genomics, unpublished). This region corresponds to amino acids 201–303 of *E. coli* CbpA, essentially comprising an isolated CTD II domain. From an alignment of the two homologues (Fig. 1B), it can be seen that the *Klebsiella* CbpA CTD II structure is an excellent model for the *E. coli* protein, with 81% sequence identity between the two molecules in this region. Importantly, none of the variable residues between the *Klebsiella* and *E. coli* proteins are located at the dimerization interface. The side chains of W287 and L290 protrude from the same side of an α helix (Fig. 10A), and form a hydrophobic surface that locates to the twofold symmetry axis of the dimer (Fig. 10B). Hydrophilic residues in the α helix (Q288, Q289, D292, Q294, S295 and S296) are not part of monomer : monomer contacts, consistent with our alanine scanning results. The α helix is flanked by a ‘tail’ at the extreme C-terminus of CbpA that appears to play little role in dimerization. Consistent with this, removal of the C-terminal 10 amino acids of CbpA, that form this tail, does not affect dimerization (Fig. 3). Interestingly, many proteins related to CbpA do not contain this C-terminal tail (Fig. 1B).

The most intriguing aspect of our analysis is the aggregation of CbpA with DNA revealed by our gel shift assays (Fig. 6) and AFM analysis (Fig. 8). Aggregation of CbpA with DNA does not appear to be influenced by the topological state of the DNA (Fig. 9) and CbpA forms aggregates with both linear and plasmid DNA (Figs 6, 9 and S4). Comparison of CbpA–DNA complexes presented here with Dps–DNA complexes previously imaged by Ceci *et al*. (2004) reveal remarkable similarities. Both proteins form large aggregates when bound to DNA and, like Dps, CbpA can protect DNA from digestion by DNase I (Fig. 9). These observations are particularly intriguing given that CbpA and Dps are both expressed in a stationary phase-specific manner, when the nucleoid undergoes a process of aggregation and compaction. However, since there is no obvious homology between Dps and CbpA, the two proteins likely interact with the DNA differently to form aggregates. Indeed, we speculate that CbpA may play a more specialized role than Dps in organization of the stationary phase chromosome; CbpA is present at lower levels than Dps and forms foci in the nucleoid whereas Dps is evenly distributed (Azam *et al*., 2000). For example, a CbpA-like protein in *Plasmodium falciparum* was recently shown to specifically interact with the chromosome replication origin (Kumar *et al*., 2010). It is also possible that CbpA acts as a ‘back-up’ for Dps in certain conditions. Consistent with previous observations we found that Fis, a major growth phase nucleoid protein, did not aggregate across the DNA but bound at specific locations (Schneider *et al*., 2001; Grainger *et al*., 2006). It is clear that these locations almost always coincide with locations at which DNA strands cross each other (Fig. 8).

In summary, CbpA was discovered as a DNA-binding protein that preferentially recognized curved DNA sequences (Yamada *et al*., 1990; Ueguchi *et al*., 1994). Subsequent studies have shown that CbpA binds to DNA with a similar affinity to other nucleoid proteins (Azam and Ishihama, 1999) and is associated with the nucleoid of starved cells (Azam *et al*., 2000). In addition to binding DNA, CbpA, by virtue of its J-domain, can act as a substitute for DnaJ in DnaK-mediated protein disaggregation. Disruption of the *cbpA* gene in a *dnaJ* genetic background results in severe growth defects at 37°C and, at permissive temperatures, these mutants have an elongated appearance and multiple nucleoids (Chenoweth *et al*., 2007). Binding of CbpA to DNA inhibits the co-chaperone activity of the protein (Chae *et al*., 2004). Moreover, association of CbpA with CbpM inhibits both the DNA-binding and co-chaperone functions of CbpA (Chae *et al*., 2004; Chenoweth *et al*., 2007). Here we show that, upon binding DNA, CbpA forms aggregates. This likely explains why CbpA is unable to function as a co-chaperone when bound to DNA. Dimerization of CbpA is required for DNA binding and we show that CbpA dimerization *in vivo* is sensitive to environmental conditions (Fig. 2B). Thus, it is also possible that an environmental signal triggers the co-chaperone activity of CbpA. We suggest that, to fully understand the biological role of CbpA, it will be essential to determine how transitions between the co-chaperone, DNA-binding and CbpM-binding activities of CbpA are controlled.

## Experimental procedures

### Strains, plasmids and oligonucleotides

Bacterial strains and plasmids are listed in Table 1. Standard techniques for recombinant DNA manipulations were used throughout. The primers listed in Table 2 were used for cloning *cbpA* and *cbpM* alleles into pKT25, pUT18, pUT18C and pET21a.

**Table 1:** Stains and plasmids.

	Description	Reference
Strains		
BTH101	F′*cya*-99 araD139 *gal*E15 *gal*K16 *rps*L1(Str^R^) *hsd*R2 *mcr*A1 *mcr*B1	Karimova *et al*. (2001)
T7 express	*fhuA2 lacZ::T7 gene1 [lon] ompT gal sulA11 R(mcr-73::miniTn10–Tet^S^)2 [dcm] R(zgb-210::Tn10–Tet^S^) endA1*Δ*(mcrC-mrr)114::IS10*	(Invitrogen)
JCB387	Δ*nir*Δ*lac*	Page *et al*. (1990)
Plasmids		
pKT25	Encodes *B. pertussis* cya gene T18 fragment and Amp^R^	Karimova *et al*. (1998)
pUT18	Encodes *B. pertussis* cya gene T18 fragment and Kan^R^	Karimova *et al*. (1998)
pUT18C	Derivative of pUT18 that allows proteins to be fused to T18 via there C-terminus	Karimova *et al*. (1998)
pET21a	T7 Expression vector containing 6×His tag	(Novagen)
pSR*fis*	pBR322-derived plasmid containing an EcoRI–HindIII *fis* fragment upstream of the *λoop* transcription terminator	This work

**Table 2:** Oligonucleotide primers.

Name	Sequence	Description
*CbpA* over expression and purification		
*cbpA*pET up	5′-catatggctagcgccttaaaggattattacgccatc-3′	Used to clone *cbpA* into pET21a
cbpApET down	5′-ggtggtgctcgagttatgctttcccccaatctttac-3′	Used to clone *cbpA* into pET21a
*cbpA*Δ10pET down	5′-ggtggtgctcgagttaagacgactgggcgtctgccagttgct-3′	Used to clone *cbpA*Δ10 into pET21a
*cbpA*Δ10 WL287 pET down	5′-ggtggtgctcgagttaagacgactgggcgtctgccagttgctgcaacagcgcggcagtg-3′	Used to clone *cbpA*Δ10 WL287 into pET21a
*cbpA*Δ50pET down	5′-ggtggtgctcgagttatttgcctttaacgcgcaatcgttgcc-3′	Used to clone *cbpA*Δ50 into pET21a
Bacterial 2-hybrid experiments		
pKT25CbpA up	5′-gcatgcctgcagggatggaattaaaggattattac-3′	Used to clone *cbpA* into pKT25
pUT18CbpA up	5′-gcatgcctgcaggatggaattaaaggattatta-3′	Used to clone *cbpA* into pUT18
pUT18CbpA down	5′-tacttaggtacccgtgctttcccccaatctttacg-3′	Used to clone *cbpA* into pUT18 and pKT25
pUT18CCbpM up	5′-gcatgcctgcaggatggctaatgttacggtgacttttac-3′	Used to clone *cbpM* into pUT18C
pUT18CCbpM down	5′-tacttaggtacccgcggatgagctacaaaccgggaaagcc-3′	Used to clone *cbpM* into pUT18C
pUT18CbpAΔ10 down	5′-tacttaggtacccgagacgactgggcgtctgccag-3′	Used to clone *cbpA*Δ10 into pUT18 or pKT25
pUT18CbpAΔ20 down	5′-tacttaggtacccgcagcgcggcagtgttttcatccgg-3′	Used to clone *cbpA*Δ20 into pUT18 or pKT25
pUT18CbpAΔ30 down	5′-tacttaggtacccgcggcatcacgattttcagtaccgc-3′	Used to clone *cbpA*Δ30 into pUT18 or pKT25
pUT18CbpAΔ40 down	5′-tacttaggtacccgatcgccggtctgttttttgctcacc-3′	Used to clone *cbpA*Δ40 into pUT18 or pKT25
pUT18CbpAΔ50 down	5′-tacttaggtacccgtttgcctttaacgcgcaatcg-3′	Used to clone *cbpA*Δ50 into pUT18 or pKT25
pUT18CbpAΔ10 S296A down	5′-tacttaggtacccgagccgactgggcgtctgccagttg-3′	Introduces S296A mutation into cbpAΔ10
pUT18CbpAΔ10 S295A down	5′-tacttaggtacccgagacgcctgggcgtctgccagttg-3′	Introduces S295A mutation into cbpAΔ10
pUT18CbpAΔ10 Q294A down	5′-tacttaggtacccgagacgacgcggcgtctgccagttgctgc-3′	Introduces Q294A mutation into cbpAΔ10
pUT18CbpAΔ10 D292A down	5′-tacttaggtacccgagacgactgggcggctgccagttgctgccac-3′	Introduces D292A mutation into cbpAΔ10
pUT18CbpAΔ10 L290A down	5′-tacttaggtacccgagacgactgggcgtctgccgcttgctgccacagcgcg-3′	Introduces L290A mutation into cbpAΔ10
pUT18CbpAΔ10 Q289A down	5′-tacttaggtacccgagacgactgggcgtctgccagtgcctgccacagcgcggcag-3′	Introduces Q294A mutation into cbpAΔ10
pUT18CbpAΔ10 Q288A down	5′-tacttaggtacccgagacgactgggcgtctgccagttgcgcccacagcgcggcagtgtt-3′	Introduces Q288A mutation into cbpAΔ10
pUT18CbpAΔ10 W287A down	5′-tacttaggtacccgagacgactgggcgtctgccagttgctgcgccagcgcggcagtgttttc-3′	Introduces W287A mutation into cbpAΔ10
W287 scramble	5′-tacttaggtacccgagacgactgggcgtctgccagttgctgnnncagcgcggcagtgttttc-3′	Randomly mutagenizes codon 287 of *cbpA*
L290 scramble	5′-tacttaggtacccgagacgactgggcgtctgcnnnttgctgccacagcgcggcagtgttttc-3′	Randomly mutagenizes codon 290 of *cbpA*
Other		
*fis up*	5′-ggctgcgaattccctggatctttcgggaaatccag-3′	Used to generate DNA fragment for EMSA
*fis down*	5′-cgcccgaagcttcatagttctgtcagctcttt-3′	Used to generate DNA fragment for EMSA

### β-Galactosidase assays

We determined β-Galactosidase levels in overnight cultures of BTH101 carrying derivatives of pKT25 and pUT18 by the Miller (1972) method. Activities are shown in Miller units and are the average of three or more independent experiments. Unless stated otherwise, all assays were done using cells grown in MacConkey broth. Note that MacConkey broth contains neutral red indicator dye that turns yellow in colour in alkaline solutions. Because β-galactosidase assays are terminated by adding NaCO_3_, we washed cells from overnight cultures thoroughly using PBS (by alternate centrifugation and resuspension steps) prior to beginning the assay.

### Overexpression and purification of CbpA and derivatives

DNA fragments encoding different *cbpA* alleles were generated using the primers listed in Table 2. After digestion with appropriate restriction enzymes these DNA fragments were cloned into the vector pET21a. To overexpress CbpA and different variants we grew 500 ml cultures of T7 express cells, carrying the appropriate pET21a derivative, to an OD_650_ of ∼0.5 at 37°C. Protein overexpression was then induced with 1 mM IPTG for a 1 h. Cells were harvested by centrifugation, resuspended in buffer FB (Grainger *et al*., 2008), and then lysed by sonication. Cell debris was removed by centrifugation and the cell lysate was loaded onto a Heparin-Sepharose column. The CbpA protein was eluted from the column using a NaCl gradient and peak fractions contained CbpA at > 95% purity as adjudged by SDS-PAGE analysis. Protein purified in this was used for gel shift assays and glutaraldehyde cross-linking assays. A further purification step, using a QFF column, removed any contaminants prior to AFM analysis.

### Electrophoretic mobility shift assays

DNA fragments for EMSA experiments were generated by PCR amplification using the appropriate DNA primers (Table 2) and *E. coli* K-12 genomic DNA as a template. PCR products were purified, cut with HindIII and EcoRI and end-labelled using [γ-^32^P]-ATP and polynucleotide kinase. DNA fragments were then incubated with purified CbpA, or the appropriate derivative, in buffer containing 20 mM Tris pH 7, 10 mM MgCl2, 100 µM EDTA, 120 mM KCl. Reactions were loaded onto a 5% polyacrylamide gel, run in 0.5x TBE at 200 V for 2–4 h and analysed as described above. EMSA experiments with unlabelled plasmid DNA were done in the absence of competitor DNA and analysed by agarose (1%) gel electrophoresis and ethidium bromide staining.

### Glutaraldehyde cross-linking

We followed the protocols of Westblade (2004). Briefly, 5 ng of purified protein was incubated with glutaraldehyde, at the indicated concentration, in 50 µl bicine buffered reactions. After 20 min reactions were stopped by adding 20 µl of ethanolamine. Proteins were then precipitated with ice-cold acetone, recovered by centrifugation, resuspended in SDS-PAGE gel-loading dye and analysed by electrophoresis.

### DNase I protection assays

Purified plasmid DNA (77 ng) was pre-incubated with CbpA or BSA, at 37°C, in DNase I reaction buffer (NEB) in a final volume of 10 µl. After 10 min 1 µl of a freshly prepared 1:200 dilution of DNase I was added. Reactions were then returned to 37°C for a further 5 min. The reactions were stopped by adding 2 µl of 10% SDS and by incubation at 75°C for 5 min. Gel-loading dye was then added and reactions were analysed by electrophoresis on 1% agarose gels.

### Atomic force microscopy

The pSR plasmid DNA for AFM analysis was isolated from cultures of *E. coli* JCB387 using a Qiagen maxiprep kit and was resuspended in water. The CbpA protein was purified as described above and Fis was purified as described previously (Grainger *et al*., 2008). Proteins and DNA were mixed at the indicated concentrations in 30 µl reactions in 1 × AFM buffer [50 mM KCl, 20 mM Hepes (pH 8.0), 2 mM NiCl_2_, and 0.005% Tween 20]. After a short incubation period (2–5 min) 20 µl of the reaction was applied to a freshly cleaved mica surface. Images were then acquired in liquid using a SNL (silicon-tip on nitride lever) AFM probe (Veeco; spring constant 0.32 N m^−1^) and a Veeco Multimode AFM with a Nanoscope IIIa controller operated in tapping mode at scan rates between 1.5 and 2.0 Hz to help minimize tip-sample interactions. The scan size was 1.5 µm^2^ at a resolution of 512 × 512 pixels. Raw data were selected with the Nanoscope software. NanoScope v5.30r3 software was used to ‘flatten’ AFM images with 3rd order polynomial fitting. AFM images were analysed further with WSxM 4.0 software (Nanotec Electronica, Spain). The height of particles was rounded to the nearest 0.2 nm. We note that buffer conditions for AFM analysis and bulk *in vitro* DNA-binding assays do, by necessity, differ substantially. Most notably, it is a prerequisite of AFM analysis to ensure that DNA molecules adhere properly to the mica surface. This is most frequently achieved by adding NiCl_2_ to reactions. We tested numerous DNA-binding proteins and found that, without exception, all had a higher affinity for DNA in standard AFM buffers. However, the properties of the protein–DNA complexes (with respect to their electrophoretic mobility) were unchanged (Fig. S5).
