# Supplementary material for: Dimerization and DNA-dependent aggregation of the Escherichia coli nucleoid protein and chaperone CbpA
Source: Mol Microbiol. 2010 Jul 30;77(5):1289–300. doi: 10.1111/j.1365-2958.2010.07292.x (PMC2955964; doi:10.1111/j.1365-2958.2010.07292.x)
Supplement: Supplementary file 1 [file mmi0077-1289-SD1.pdf]

Figure S1

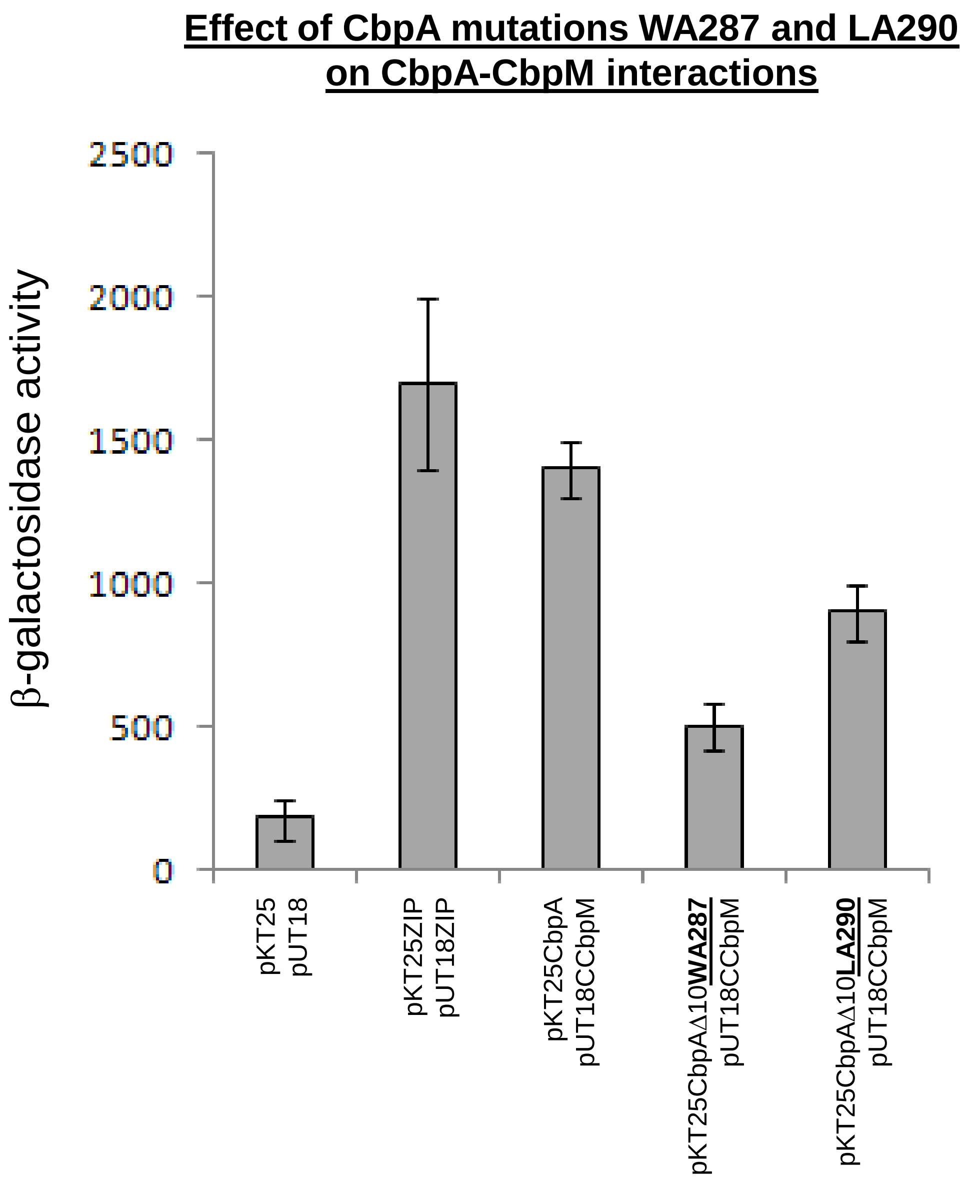

Figure S1: Dimerisation defective CbpAΔ10 derivatives with reduced affinity for CbpM. The figure shows β-galactosidase activities for BTH101 cells carrying derivatives of pKT25 and pUT18. Plasmids encoding CbpAΔ10 with alanine substitutions at amino acids 287 or 290 are highlighted with boldtype face and are underscored.

Figure S2

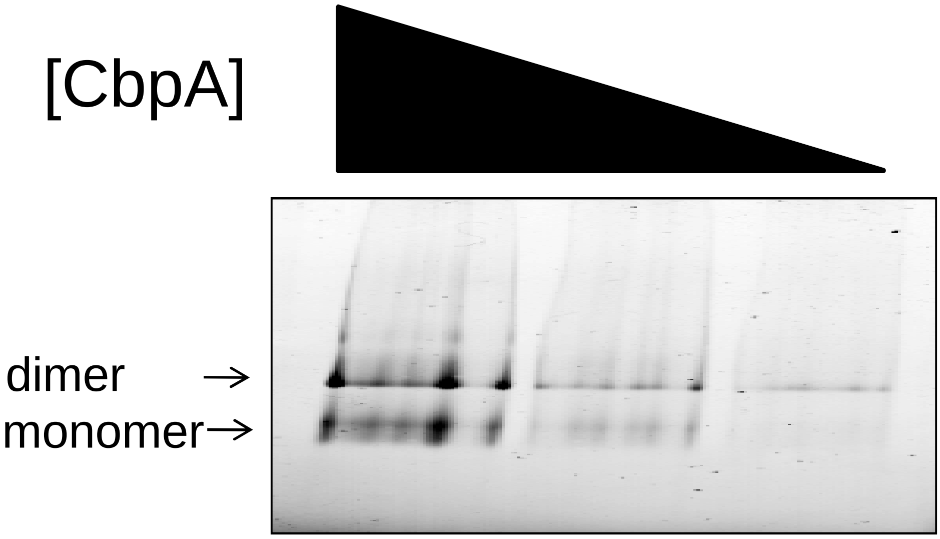

Figure S2: Detection of CbpA-CbpA interactions by native gel analysis. The figure shows different concentrations of purified CbpA protein run on a native poylacrylamide gel.

Figure S3

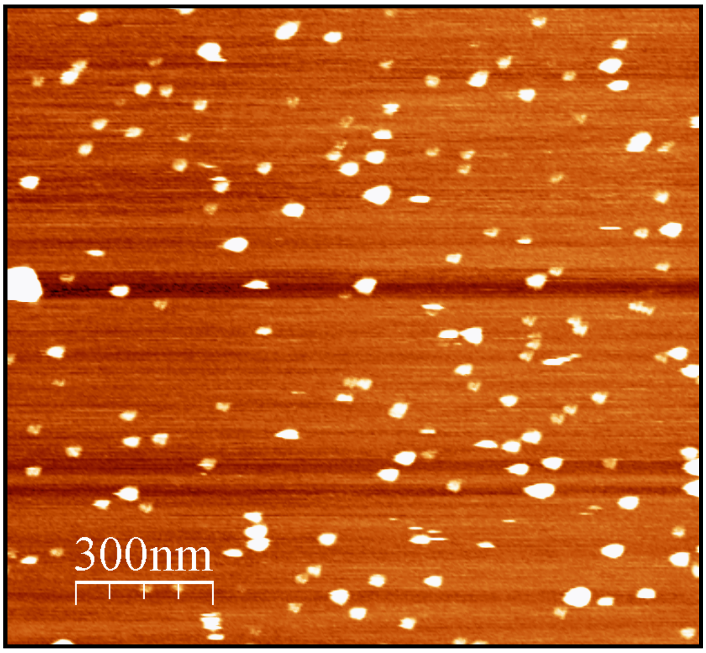

Figure S3: Complexes formed between CbpA and plasmid DNA using excess CbpA. The figure shows an AFM image of a 1.5 μm x 1.5 μm region of a freshly cleaved 0.5 cm^2 mica surface. The surface was pre-incubated with a 20 μl solution of plasmid DNA (23 ng) in the absence of 1 μM CbpA.

Figure S4

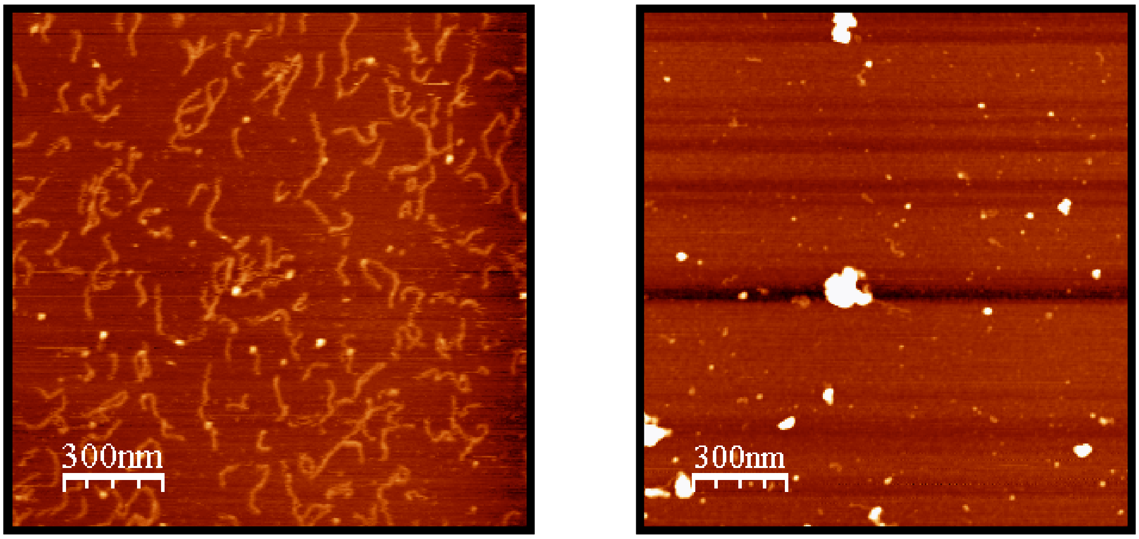

Figure S4: Complexes formed between CbpA and linear DNA visualised by AFM. A) DNA only B) DNA and CbpA.

Figure S5

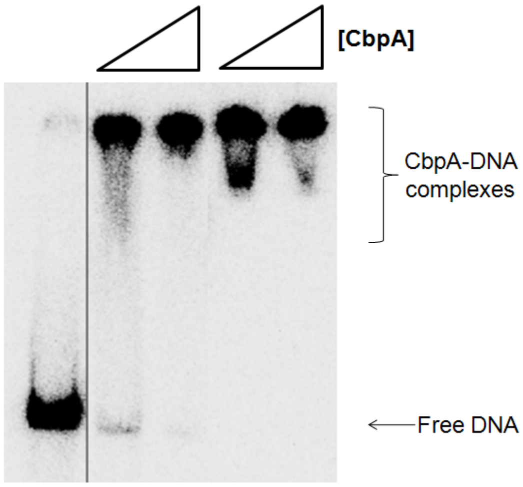

Figure S5: Comparison of interactions between CbpA and DNA in “EMSA” and “AFM” buffers.
